# Promotion of Both Proliferation and Neuronal Differentiation in Pluripotent P19 Cells with Stable Overexpression of the Glutamine Transporter slc38a1

**DOI:** 10.1371/journal.pone.0048270

**Published:** 2012-10-24

**Authors:** Masato Ogura, Takami Kakuda, Takeshi Takarada, Noritaka Nakamichi, Ryo Fukumori, Yeong-Hun Kim, Eiichi Hinoi, Yukio Yoneda

**Affiliations:** 1 Laboratory of Molecular Pharmacology, Kanazawa University Graduate School, Kanazawa, Japan; 2 R & D Division, Itoen Ltd., Makinohara City, Japan; Rutgers University, United States of America

## Abstract

**Background:**

We previously demonstrated the functional expression in newborn rat neocortical astrocytes of glutamine transporter (GlnT = slc38a1) believed to predominate in neurons over astroglia in the brain. In order to evaluate the possible role of this transporter in neurogenesis, we attempted to establish stable transfectants of GlnT in mouse embryonal carcinoma P19 cells endowed to proliferate for self-renewal and differentiate into progeny cells such as neurons and astroglia, in addition to *in vitro* pharmacological profiling of the green tea ingredient theanine, which is shown to be a potent inhibitor of glutamine transport mediated by GlnT in cultured neurons and astroglia.

**Methodology/Principal Findings:**

The full-length coding region of rat GlnT was inserted into a vector for gene transfection along with selection by G418, followed by culture with all-*trans* retinoic acid under floating conditions and subsequent dispersion for spontaneous differentiation under adherent conditions. Stable overexpression of GlnT led to marked increases in the size of round spheres formed during the culture for 4 days and 3-(4,5-dimethyl-2-thiazolyl)-2,5-diphenyl-2H-tetrazolium bromide reduction, with concomitant promotion of subsequent differentiation into cells immunoreactive for a neuronal marker protein. In these stable GlnT transfectants before differentiation, drastic upregulation was seen for mRNA expression of several proneural genes with a basic helix-loop-helix domain such as NeuroD1. Although a drastic increase was seen in NeuroD1 promoter activity in stable GlnT transfectants, theanine doubled NeuroD1 promoter activity in stable transfectants of empty vector (EV), without affecting the promoter activity already elevated in GlnT transfectants. Similarly, theanine promoted cellular proliferation and neuronal differentiation in stable EV transfectants, but failed to further stimulate the acceleration of both proliferation and neuronal differentiation found in stable GlnT transfectants.

**Conclusions/Significance:**

GlnT would promote both proliferation and neuronal differentiation through a mechanism relevant to the upregulation of particular proneural genes in undifferentiated P19 cells.

## Introduction

Glutamine (Gln) is believed to play a dual role as a precursor for amino acid neurotransmitters, such as glutamate and γ-aminobutyric acid (GABA), in the central nervous system [1], and as a principal substrate for the Gln transporter (GlnT) ( = slc38a1; also referred to as SAT1/SNAT1/ATA1) shown to highly predominate in neurons over other cells in the brain. In astrocytes, the excitatory neurotransmitter glutamate is converted by the catalytic action of Gln synthase to Gln that is released to extracellular spaces, and then extracellular Gln is incorporated through GlnT expressed by neurons toward the synthesis of glutamate by glutaminase to fuel the neurotransmitter pool of glutamate at glutamatergic nerve terminals as the glutamate/Gln cycle [1]–[3]. Glutamine is actively incorporated into intracellular spaces in rat brain slices [4], indeed, while several independent lines of evidence indicate the involvement of different amino acid transporters in mechanisms underlying the active transmembrane incorporation process of Gln in the brain. For example, Gln transport is mediated by at least three sodium-dependent transport systems in the brain. These include system-A [5]–[7], system-ASC [8], [9] and system-N [10]. Moreover, Gln is transported through the sodium-independent system-L carrier composed of heteromeric assemblies of different subunits [2], [11], [12]. Of these different transporters capable of incorporating extracellular Gln across plasma membranes, the system-A transporter GlnT is supposed to be exclusively localized in neurons with high and selective affinity for Gln in the brain [13]–[15].

However, recent studies have demonstrated the expression of GlnT mRNA in primary cultured astrocytes prepared from rat whole brain, in addition to [^3^H]Gln influx and efflux activities insensitive to the selective system-A transporter inhibitor N-methylaminoisobutyric acid (MeAIB) [16], [17]. Double immunohistochemistry analysis has demonstrated a clear co-localization of immunoreactivities for GlnT and a glial marker protein, glial fibrillary acidic protein, in cerebral cortex of adult rat and human brains [18]. We have shown that GlnT is functionally expressed in cultured rat neocortical astrocytes devoid of neuronal marker expression, as well as cultured rat neocortical neurons, along with [^3^H]Gln incorporation sensitive to MeAIB [19]. Moreover, GlnT mRNA expression is down-regulated by lipopolysaccharide through the decreased promoter activity [19] and up-regulated through the transactivation mediated by cAMP/protein kinase A signals [20] in cultured astrocytes, respectively. The adenylyl cyclase activator forskolin is shown to increase the steady-state levels of mRNA for both GlnT (slc38a1) and ATA2 (slc38a2) isoforms with concomitant facilitation of sodium-dependent accumulation of [^3^H]MeAIB in the human liver cell line HepG2 [21].

**Table 1:** Primers used for RT-PCR in this study.

Genes	Upstream (5′-3′)	Downstream (5′-3′)
**bHLH transcriptional factors**		
Mash1	AACAAACCAGACAGCCAACC	AAAGGCTGTCCGAGAACTGA
Math3	TCTTCGACTGGCAAGGAACT	ACTAATGCTCAGGGGTGGTG
NeuroD1	CAAAGCCACGGATCAATCTT	CCCGGGAATAGTGAAACTGA
Hes1	GCCAATTTGCCTTTCTCATC	AGGCGCAATCCAATATGAAC
Hes5	ATGCTCAGTCCCAAGGAGAA	CGCTGGAAGTGGTAAAGCAG
Gapdh	ACCACAGTCCATGCCATCAC	TCCACCACCCTGTTGCTGTA

On the other hand, the green tea ingredient theanine ( = γ-glutamylethylamide) is a structural analog of Gln and shown to inhibit [^3^H]Gln incorporation in a concentration-dependent manner in cultured rat neocortical astrocytes and neurons, which both exhibit activities to incorporate [^3^H]theanine as well as [^3^H]Gln [22]. Theanine elicits a suppression of the stimulation by caffeine of brain excitability [23] along with a reduction of the systemic blood pressure [24]. Prior intracerebroventricular injection of theanine leads to the protection of the hippocampal CA1 pyramidal neurons from delayed neuronal cell death in gerbils with bilateral forebrain global ischemia [25], furthermore, while theanine is a poor inhibitor of ligand binding to three different ionotropic glutamate receptor subtypes in rat cortical neurons [26]. By contrast, both neurons and astroglia are derived from neural stem cells defined as primitive cells endowed to proliferate for self-renewal and to differentiate into different progeny lineages [27]–[31]. These previous findings thus led us to establish stable transfectants of GlnT in mouse embryonal carcinoma P19 cells endowed to proliferate for self-renewal and differentiate into progeny cells, such as neurons and astroglia, in addition to evaluating *in vitro* pharmacological profiles of theanine on their proliferation and differentiation activities.

**Figure 1:**
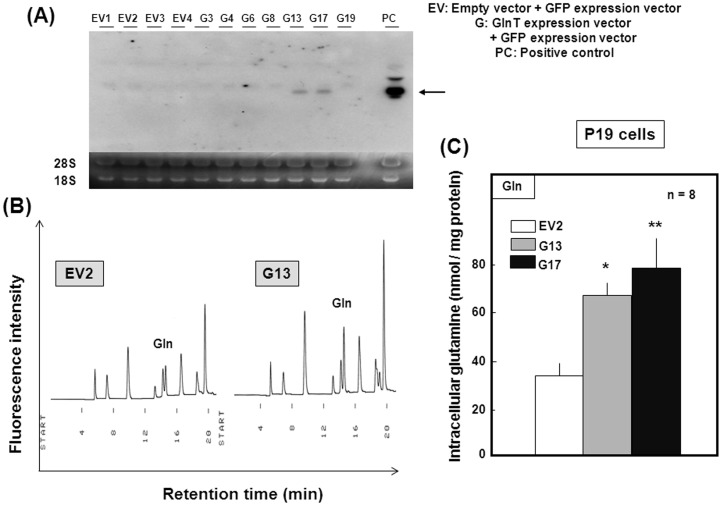
Establishment of stable GlnT transfectants in pluripotent P19 cells. (A) P19 cells were stably transfected with GlnT expression vector, followed by selection of adequate clones after culture with G418 and subsequent determination of GlnT mRNA by Northern blotting. (B) Both EV2 and G13 clones were homogenized and subjected to determination of endogenous levels of Gln by HPLC. (C) Endogenous levels of Gln were quantified by HPLC in EV2, G13 and G17 clones (*n* = 8). *P<0.05, **P<0.01, significantly different from the control value in EV2 cells.

## Materials and Methods

### Materials

Mouse embryonal carcinoma P19 cells were purchased from Riken Cell Bank (Tsukuba, Japan). Theanine was purchased from Tokyo Kasei Kogyo (Tokyo, Japan). Antibodies against microtubules-associated protein-2 (MAP2) and β-tubulin, Hoechst33342, 3-(4,5-dimethyl-2-thiazolyl)-2,5-diphenyl-2H-tetrazolium bromide (MTT) and all-*trans*-retinoic acid (ATRA) were purchased from Sigma Chemical Company (St. Louis, MO, USA). An Enhanced Chemiluminescence detection kit was purchased from Amersham Biosciences (Piscataway, NJ, USA). An anti-mouse IgG antibody conjugated with rhodamine and an anti-rabbit IgG antibody conjugated with fluorescein were obtained from ICN Pharmaceuticals (Aurora, OH, USA). Dulbecco’s modified Eagle’s medium (DMEM), alpha minimal essential medium (αMEM), Opti-MEM and fetal bovine serum (FBS) were purchased from GIBCO BRL (Grand Island, NY, USA). Epidermal growth factor (EGF) was provided by Biomedical Technologies (Stoughton, MA, USA). M-MLV Reverse Transcriptase, Lipofectamine reagent, Plus reagent and G418 were supplied by Invitrogen (San Diego, CA, USA). The dual luciferase assay system and pRL-SV40 were purchased from Promega (Madison, WI, USA). ISOGEN was purchased from NIPPON GENE CO. (Tokyo, Japan). rTaq DNA polymerase was obtained from TAKARA BIO INC. (Otsu, Japan). All other chemicals used were of the highest purity available. Culture plates (1.9 cm^2^, 24 wells) were purchased from Nalge Nunc International (Rochester, NY, USA).

**Figure 2:**
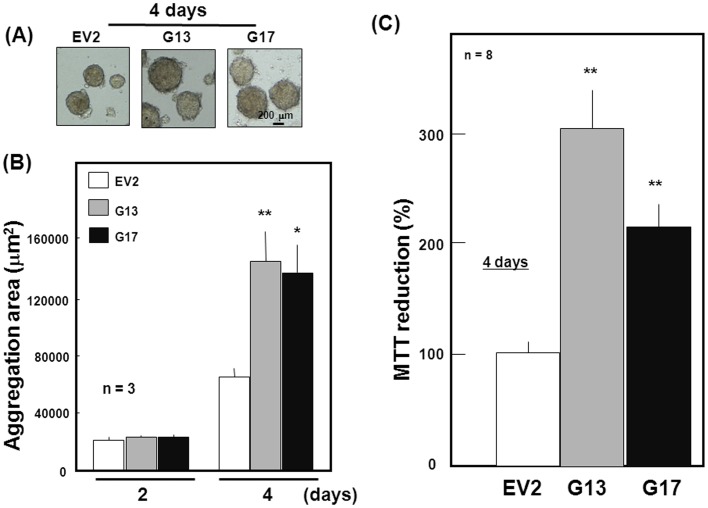
Proliferation of stable transfectants. (A) EV2, G13 and G17 clones were cultured with ATRA under floating conditions for 4 days. Typical phase contrast pictures are shown in this panel. (B) Transfectant clones were cultured with ATRA under floating conditions for a period up to 4 days, followed by calculation of the size of spheres formed on Day 2 and Day 4 in five different visual fields chosen at random in a blinded fashion (*n* = 3). (C) Cells were cultured for 4 days, followed by determination of MTT reduction (*n* = 8). *P<0.05, **P<0.01, significantly different from each control value in EV2 cells.

### Establishment of Stable Transfectants

The expression vector of GlnT (pSI-GlnT) was prepared from the product by RT-PCR of rat GlnT coding region in primary cultured cortical neurons using a specific GlnT primer. This product was inserted into pSI, which is a vector for gene transfection, by using the Ligation High regent as described previously [32]. Mouse embryonal carcinoma P19 cells were plated at a density of 1.5×10^5^ cells/cm^2^, followed by culture in DMEM with 10% FBS for 24 h and subsequent stable transfection with pSI- GlnT, pSI-GFP and pcDNA3.1 vectors, or pSI, pSI-GFP and pcDNA3.1 vectors, using 2 µg of DNA and Lipofectamine and Plus reagent in 0.5 ml of Opti-MEM described previously [33]. After 24 h, and every 48 h thereafter for 2 weeks, culture medium was replaced with fresh medium containing 500 µg/ml of G418. Pools of 17 clones were isolated as stable transfectants with GlnT expression vector and an empty vector (EV) in P19 cells for further studies using clones between passages 3 and 6.

### Northern Blotting Analysis

GlnT cDNA including entire coding region was prepared from rat cortical neuronal mRNA by RT-PCR as described previously [32]. cDNA was then subcloned into pT7 blue vector. Plasmids were linearized and transcriptionally labeled by T7 RNA polymerase to make an antisense digoxigenin (DIG)-labeled cRNA probe. The DIG-labeled cRNA was stored at −80°C until use. An aliquot of total RNA (5 µg/lane) was subjected to electrophoresis on a 1% denatured agarose gel containing 2.2 M formaldehyde at a constant voltage of 50 V at room temperature and subsequently transferred onto positively charged nylon transfer membranes by capillary blotting. After fixation of RNA to the blot by UV crosslinking, blotted membranes were prehybridized at 68°C for 1 h, and subsequently hybridized with a denatured DIG-labeled RNA probe of GlnT at 68°C for 16 h. The hybridized membrane was successively washed with 2×SSC containing 0.1% sodium dodecylsulfate (SDS) at room temperature for 5 min twice and 0.1×SSC containing 0.1% SDS at 68°C for 15 min twice. The membrane was incubated with anti-DIG-AP-Fab, followed by incubation with CDP-star and subsequent exposure to X-ray films for appropriate periods to detect chemiluminescence. Hybridization signals were detected by autoradiography [34].

**Figure 3:**
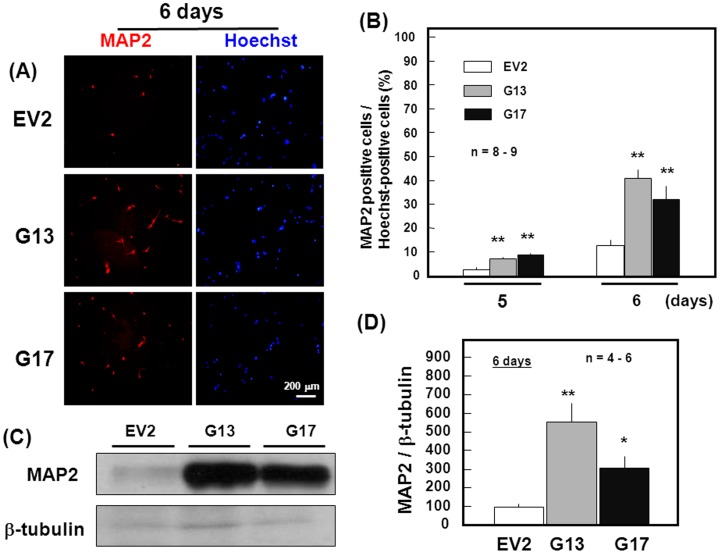
Differentiation of stable transfectants. Cells were dispersed after the culture with ATRA for 4 days, followed by further culture for an additional 2 days and subsequent immunocytochemical detection of MAP2 together with Hoechst33342 staining (*n* = 8–9). Typical pictures are shown in the panel (A), while quantitative data are shown in the panel (B) for cells cultured for 5 and 6 days in total. Transfectant clones were also cultured with ATRA for 4 days, followed by dispersion for further culture for an additional 2 days and subsequent immunoblotting analysis on MAP2 (*n* = 4–6). Typical pictures are shown in the panel (C), while quantitative data are shown in the panel (D). *P<0.05, **P<0.01, significantly different from each control value in EV2 cells.

### High Performance Liquid Chromatography (HPLC)

Cells were washed in cold PBS, followed by homogenization in 0.2 M perchrolic acid with a sonicator and subsequent centrifugation at 20,000 g for 10 min. An aliquot of supernatants was neutralized with 1 M KHCO_3_, followed by centrifugation and subsequent reaction for 2 min at room temperature with a derivatization reagent [5 mg/ml o-phthalaldehyde, 1% β-mercaptoethanol and 0.36 M potassium borate (pH 10.4)] for HPLC analysis as described previously [32]. An aliquot was injected into an analysis column (Symmetry C_18_ 3.5 µm, 4.6×75 mm). Elution was conducted using a programmable pump (Shimadzu LC-9A) with a linear gradient to 70% acetonitrile and 20 mM sodium phosphate (pH 6.0) at a flow rate of 1.0 ml/min. Derivatized amino acids were detected with a fluorometer (Shimadzu RF-10XL) by using an excitation wavelength of 340 nm and an fluorescence wavelength of 450 nm.

### Determination of Cellular Proliferation

Five different visual fields were chosen at random from each culture well with undifferentiated P19 cells cultured with ATRA for a period of up to 4 days as described below under a phase contrast micrograph in a blinded fashion, followed by calculation of areas of round aggregates composed of clustered cells in parallel experiments for summation using Scion Image β 4.02 software (Scion Co., Frederick, MD, USA). Moreover, cultured cells collected by centrifugation at 300 g were incubated with 0.5 mg/ml MTT in phosphate-buffered saline (PBS) for 1 h at 37°C, followed by addition of 0.04 M HCl in isopropanol and subsequent shaking of the mixture for 10 min to dissolve the formazan. The dissolved suspension was subjected to an ELISA reader and the absorbance at a wavelength of 550 nm was measured as described previously [35].

**Figure 4:**
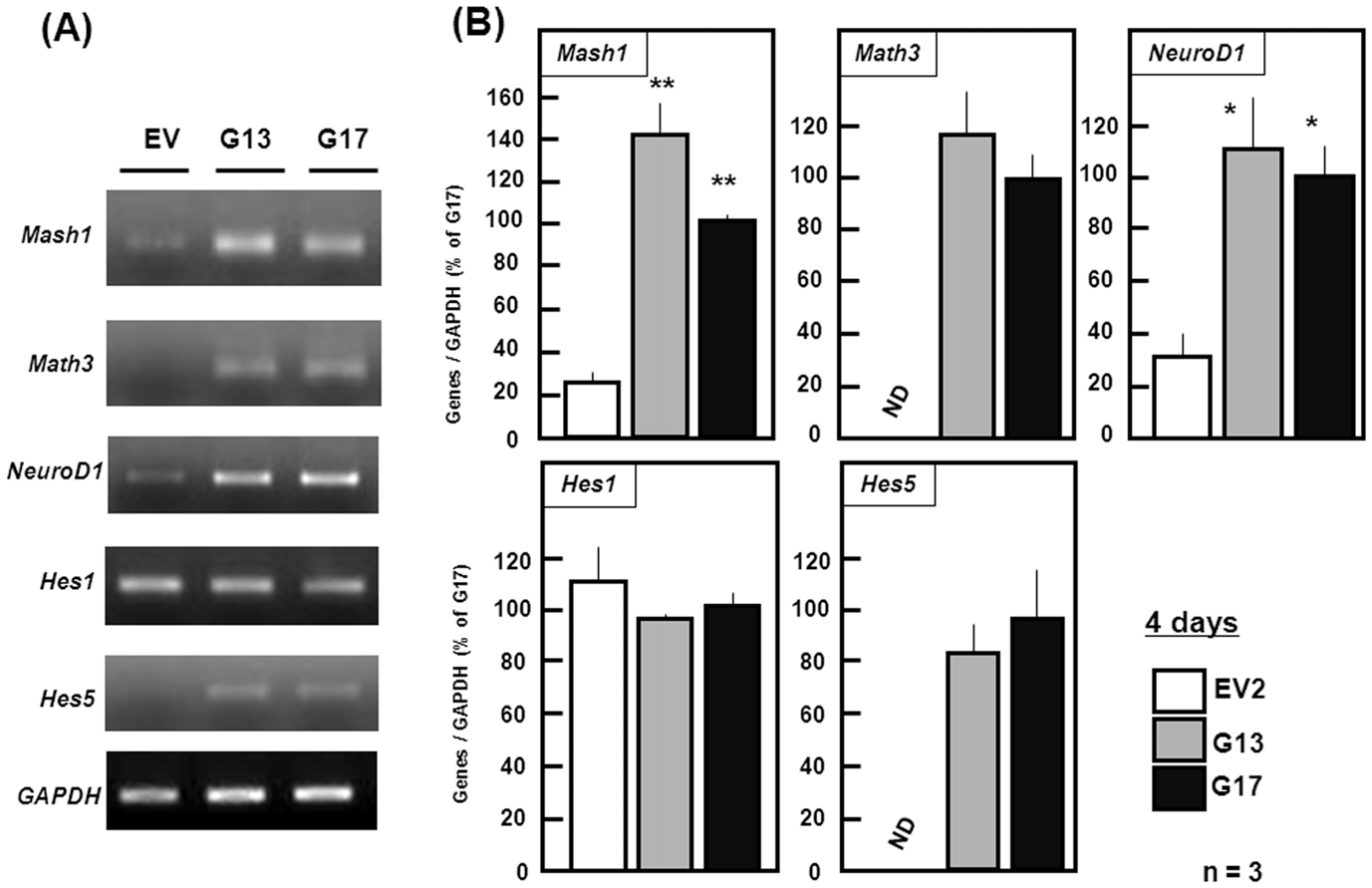
Proneural genes with bHLH in stable transfectants. Transfectants were cultured with ATRA for 4 days under floating conditions, followed by cell harvest for total RNA extraction and subsequent RT-PCR analysis (*n* = 3). Typical pictures are shown in the panel (A), while quantitative data are shown in the panel (B). Values represent percentages of the ratios of expression of a proneural gene to GAPDH in each stable transfectant over those in G17 transfectant cells. *P<0.05, **P<0.01, significantly different from each control value in EV2 cells. ND, not detectable.

### Determination of Cellular Differentiation

The pluripotent P19 stem cells derived from murine embryonal carcinoma are shown to differentiate into neuronal and astroglial lineages after the commitment in the presence of ATRA as described previously [36]. Undifferentiated P19 cells were plated onto 0.2% agarose coated dishes (φ100 mm) at a density of 1×10^5^ cells/ml in αMEM supplemented with 5% FBS and 0.5 µM ATRA, followed by culture for 4 days in either the presence or absence of 100 µM theanine under floating conditions for proliferation. Cell doubling time is reported to be 14.3±1.0 h with P19 cells [37]. These floating cells were harvested for trypsinization, followed by plating onto dishes previously coated with poly-L-lysine at a density of 2×10^5^ cells/ml in αMEM supplemented with 10% FBS and subsequent culture for an additional period up to 6 days in the absence of ATRA for induction of spontaneous differentiation. Culture medium was changed every 2 days, and cultures were maintained in a humidified atmosphere of 5% CO_2_/95% air at 37°C.

### Reverse-transcription Polymerase Chain Reaction (RT-PCR)

Total RNA was extracted from cultured cells using the standard ISOGEN procedure and then subjected to the synthesis of cDNA for RT-PCR. The individual cDNA species were amplified in a reaction mixture containing a cDNA aliquot, PCR buffer, dNTPs, relevant sense and antisense primers (Table 1) and rTaq DNA polymerase as described previously [38]. Reactions were initiated by incubating at 95°C for 10 min and PCR (denaturation at 95°C for 1 min, annealing at 50°C for 1.5 min and extension at 72°C for 2 min) was performed for 25 cycles with a final extension at 72°C for 5 min. Quantification was done at the cycle number with high linearity between mRNA expression and cDNA production by using primers for the housekeeping gene glyceraldehydes-3-phosphate dehydrogenase (GAPDH). In preliminary experiments, a clearly linear correlation was optimized with each primer set. The PCR product of each molecule yielded single bands corresponding to the expected base pairs. PCR reaction products were separated on 1.5% agarose gels with ethidium bromide for visualization. Appropriate PCR products were extracted from agarose gel using DNA extraction spin columns, followed by sequencing with ABI Prism 310 Genetic Analyzer (Perkin-Elmer) using a cycle sequencing kit. The relative abundance of each PCR product was determined by quantitative analysis of digital photographs of gels using Scion Image β4.02 software (Scion Co., Frederick, MD, USA). Densitometry was done with the individual PCR products by using a densitograph, followed by calculation of the ratios of expression of mRNA for each gene over that for GAPDH. For comparison of gene expression among different transfectant clones, percentages were calculated on the basis of expression ratios in G17 clone as 100%.

**Figure 5:**
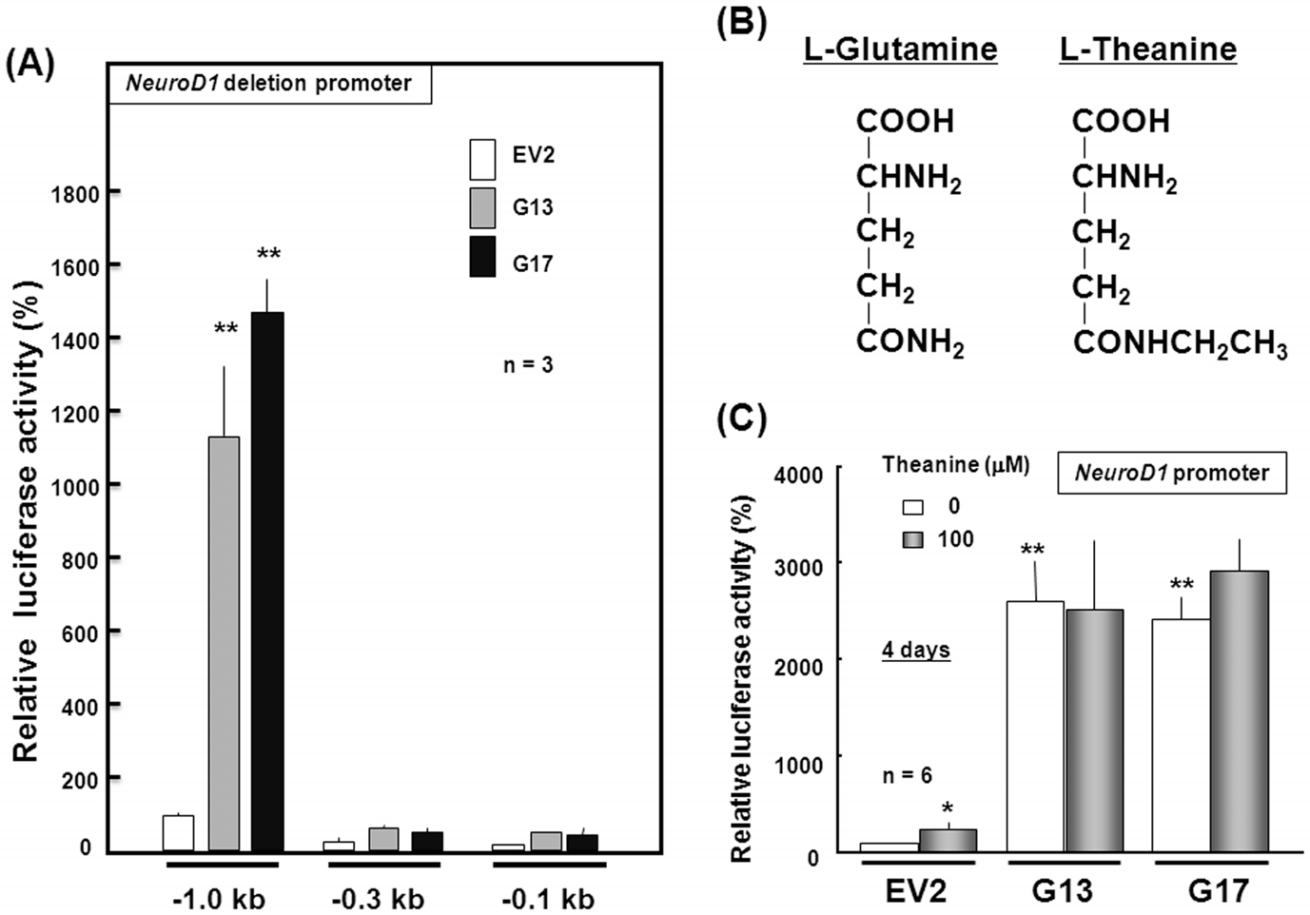
Effects of theanine on NeuroD1 promoter activity in stable transfectants. (A) Each transfectant clone was transfected with NeuroD1 reporter plasmids with different lengths, followed by further culture with ATRA for an additional 4 days and subsequent determination of luciferase activity (*n* = 3). (B) Chemical structures of L-glutamine and L-theanine. (C) Each transfectant clone was also transfected with full-length NeuroD1 reporter plasmid, followed by further culture with ATRA in either the presence or absence of 100 µM theanine for an additional 4 days and subsequent determination of luciferase activity (*n* = 6). *P<0.05, **P<0.01, significantly different from each control value obtained in EV2 cells not cultured with theanine.

**Figure 6:**
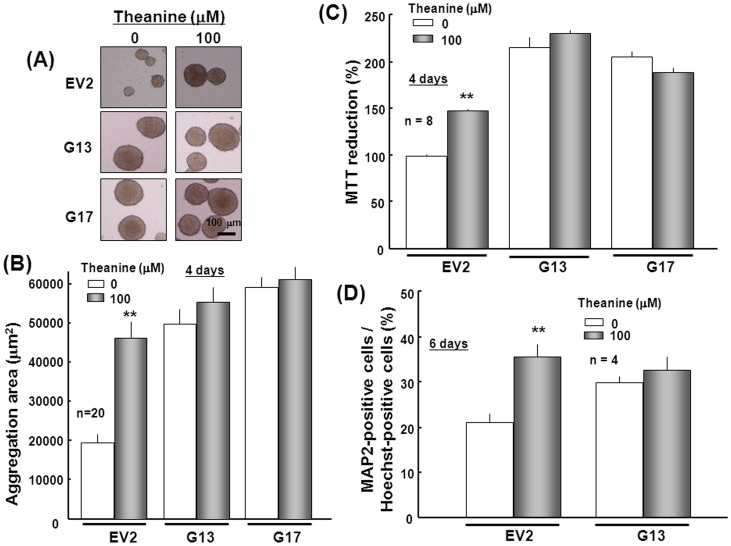
Effects of theanine on cellular proliferation and differentiation in stable transfectants. Each transfectant clone was cultured with ATRA for 4 days in either the presence or absence of 100 µM theanine. Typical phase contrast micrographs are shown in the panel (A), while quantitative data are shown for the size of spheres formed during culture (*n* = 20) in the panel (B) and for MTT reduction (*n* = 8) in the panel (C), respectively. (D) Each transfectant clone was also cultured with ATRA in either the presence or absence of 100 µM theanine for 4 days, followed by dispersion for further culture for an additional 2 days and subsequent quantitative determination of the ratio of MAP2-positive cells over Hoechst33342-positive cells (*n* = 4). **P<0.01, significantly different from each control value obtained in EV2 cells not cultured with theanine.

### Preparation of Reporter Plasmids

Reporter plasmids for NeuroD1 promoter were individually prepared as follows. Mouse NeuroD1 promoter was obtained by cloning using the forward primer 5′-TCGAGCTC (SacI site) TGAGGTCATTCATTACTCC-3′ and the reverse primer 5′-GCAAGCTT (HindIII site) GAATTCCTCGTGTCCCGG-3′ with mouse genome. NeuroD1 (−1000 to +11) promoter fragments were cloned into the promoterless pGL-3 basic vector, to create the recombinant plasmid –1000/+11 NeuroD1-LUC. The deletion mutants of these promoter plasmids (−366/+11 and −112/+11 bp upstream for NeuroD1-LUC) were made from the recombinant plasmid –1000/+11 NeuroD1-LUC by using restriction enzymes and T4 DNA polymerase, respectively.

### Reporter Assay

The pluripotent P19 cells were transiently transfected with a reporter plasmid containing the full-length or deletion mutant promoters of NeuroD1 along with the internal control vector pRL-SV40, by using the calcium phosphate method. In brief, 5 µg of plasmid DNA was dissolved in 250 µl of 0.25 M CaCl_2_, followed by addition of 250 µl of 11.76 g/l N,N-bis(2-hydroxyethyl)-2-aminoethanesulfonic acid sodium salt, 16.36 g/l NaCl, and 0.21 g/l Na_2_HPO_4_ at pH 6.95 and subsequent standing for 20 min before the addition to cultured cells drop-wise. Cells were further cultured at 35°C under 3% CO_2_ for an additional 24 h, followed by dispersion in trypsin-EDTA and subsequent suspension in αMEM containing 10% FBS for centrifugation at 400 g for 5 min. Cells were collected for plating at 1×10^5^ cells/ml in plates (φ100 mm) previously coated with 0.2% agarose, and cultured in DMEM/F-12 supplemented with 10% FBS and 0.5 µM ATRA in either the presence or absence of theanine for 4 days. Cells were lysed for determination of the luciferase activity using specific substrates in a luminometer according to the manufacturer’s protocol. Transfection efficiency was normalized by determining the activity of Renilla luciferase. Approximately 30% of cells expressed green fluorescent protein (GFP) in P19 cells transfected with the EGFP-C2 plasmid under the transfection condition employed.

### Procedures for Immunocytochemistry

Cells were washed with PBS, followed by fixation with 4% paraformaldehyde for 20 min at 4°C and subsequent blocking with 10% normal horse serum or goat serum in PBS containing 0.1% Triton-X [39]. Cells were then reacted with an antibody adequately diluted against the neuronal marker protein MAP2 overnight at 4°C. Cells were reacted with the corresponding secondary antibody, an anti-mouse IgG antibody conjugated with rhodamine. Quantification was done by counting the number of cells immunoreactive for MAP2 on immunocytochemistry analysis, followed by calculation of individual percentages over the number of total cells stained with Hoechst33342 under a fluorescence microscope.

### Western Blotting Analysis

Cultured P19 cells were washed with PBS and collected for lysis in CSK buffer [(in mM): NaCl, 100; sucrose, 300; PIPES (pH 6.8), 10; MgCl_2_, 3; EDTA, 1 and 0.5% Triton X-100]. Aliquots of extracts were added at a volume ratio of 4∶1 to 10 mM Tris-HCl buffer (pH 6.8) containing 10% glycerol, 2% SDS, 0.01% bromophenol blue and 5% 2-mercaptoethanol, followed by mixing and boiling at 100°C for 5 min. Each aliquot of 10 µg proteins was loaded on a 7.5% polyacrylamide gel for electrophoresis at a constant current of 15 mA/plate for 2 h at room temperature and subsequent blotting to a polyvinylidene fluoride membrane previously treated with 100% methanol. After blocking by 5% skimmed milk dissolved in 20 mM Tris-HCl buffer (pH 7.5) containing 137 mM NaCl and 0.05% Tween 20, the membrane was reacted with an antibody against MAP2 (1∶5000) or β-tubulin diluted with the buffer containing 1% skimmed milk to 1.0×10^4^-fold, followed by a reaction with an anti-mouse IgG antibody conjugated with peroxidase. Proteins reactive with those antibodies were detected with the aid of ECL™ detection reagents through exposure to X-ray films [40].

### Data Analysis

Quantitative data are expressed as the mean ± S.E. and the statistical significance was determined by the two-tailed Student’s *t*-test or the one-way analysis of variance (ANOVA) with Bonferroni/Dunnett *post hoc* test.

**Figure 7:**
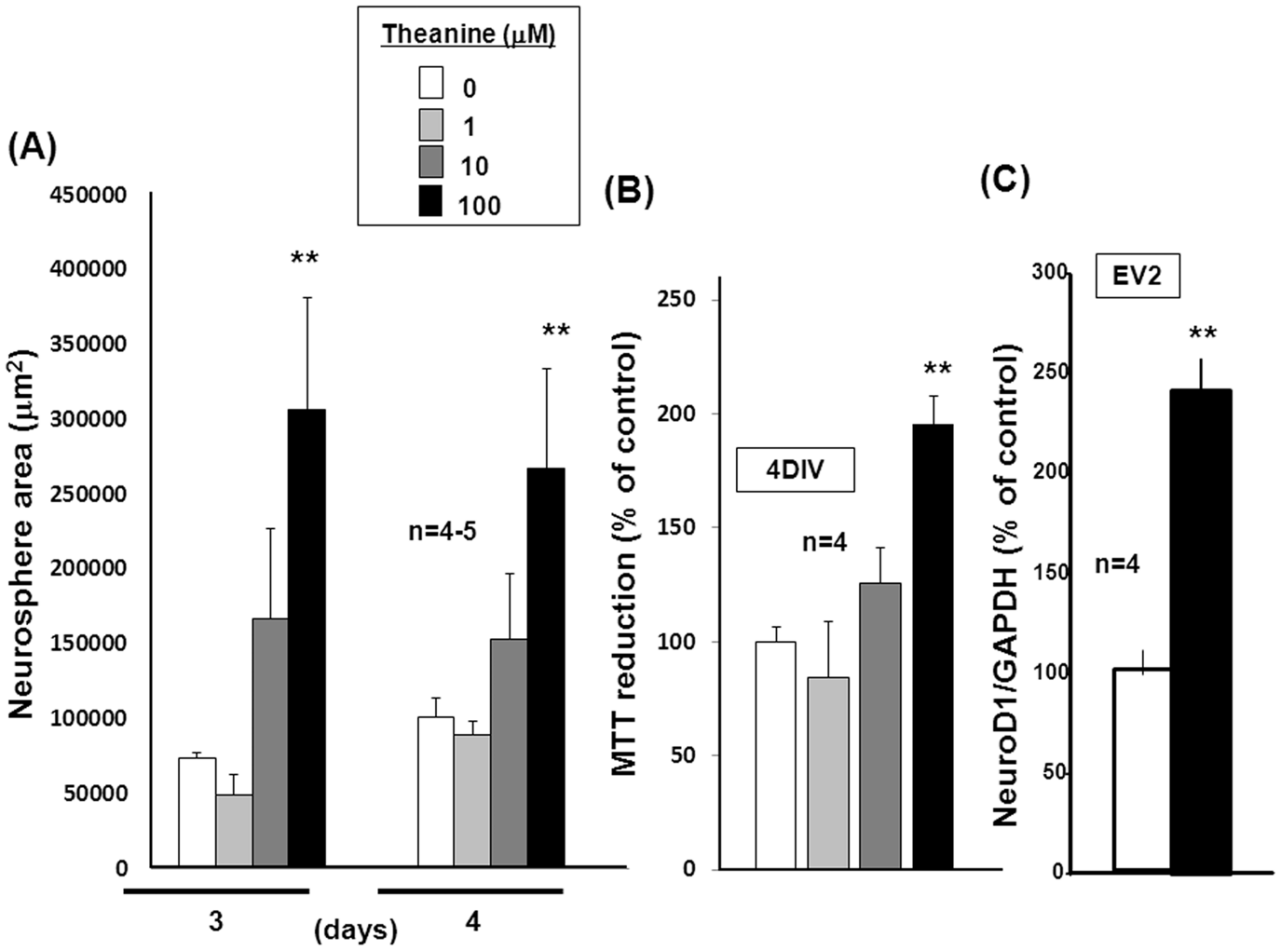
Similarity between GlnT overexpression and theanine. P19 cells were cultured with ATRA for 3 or 4 days in either the presence or absence of theanine at a concentration range of 1 to 100 µM, followed by determination of cellular proliferation activity with (A) aggregate size (*n* = 4–5) and (B) MTT reduction (*n* = 4). (C) EV2 clone was cultured with ATRA for 4 days, followed by RNA extraction and subsequent determination of NeuroD1 mRNA expression (*n* = 4). **P<0.01, significantly different from each control value obtained in cells not exposed to theanine.

## Results

### Stable GlnT Transfectants

Amongst 17 clones selected by G418 in pluripotent P19 transfectant cells, GlnT mRNA was highly detected in several clones, such as G13 and G17, on Northern blotting analysis (Fig. 1A). However, an attempt to demonstrate the overexpression of GlnT at the protein level was unsuccessful so far on Western blotting analysis using three kinds of the anti-GlnT antibody available from different commercial sources (Proteintech, 12039-1-AP; Santa Cruz Biotechnology, sc-33439; Santa Cruz Biotechnology, sc-67080). We thus determined the endogenous level of Gln in these stable transfectants. The level of endogenous Gln was higher in G13 clone cells than in EV2 cells when determined by HPLC (Fig. 1B). Quantification of these data revealed that both G13 and G17 clones of stable GlnT transfectants showed more than two times higher Gln contents compared to the stable EV2 transfectant clone (Fig. 1C).

Pluripotent P19 cells were clustered to form round aggregates with growing sizes in proportion to the culture period up to 4 days under floating conditions along with a marked increase in MTT reduction as indices of cellular proliferation. Stable GlnT transfection led to a marked increase in the size of aggregates formed after the culture with ATRA for 4 days (Fig. 2A). In both stable GlnT transfectant clones cultured for 2 days, however, no significant increase was seen in the size of aggregates despite the significantly increased size in both transfectant cells cultured for 4 days (Fig. 2B). Moreover, MTT reduction was more than doubled in both stable GlnT transfectant clones compared to stable EV transfectants when determined after the culture for 4 days (Fig. 2C).

Pluripotent P19 cells differentiated into cells immunoreactive for MAP2 within 2 to 4 days after dispersion of aggregates previously cultured for 4 days, along with the disappearance of MAP2-positive cells and subsequent appearance of cells immunoreactive for the astroglial marker protein glial fibrillary acidic protein within 12 to 16 days as reported elsewhere [41], [42]. In both stable GlnT transfectant clones cultured for an additional 2 days after dispersion, however, a marked increase was seen in the number of cells immunoreactive for MAP2 over those positive to Hoechst33342 staining compared to stable EV transfectants (Fig. 3A). Repetition of these experiments for quantification clearly revealed that stable GlnT transfection significantly increased the number of MAP2-positive cells over that of Hoechst33342-positive cells in P19 cells cultured for 5 to 6 days in total (Fig. 3B). In GlnT transfectant cells cultured for 6 days, a marked increase was invariably seen in MAP2 expression over EV transfectants on Western blotting analysis (Fig. 3C). Stable GlnT overexpression was effective in more than tripling MAP2 levels in P19 cells cultured for 6 days (Fig. 3D).

### Expression of Proneural Genes

An attempt was next made to determine whether stable overexpression of GlnT leads to altered expression of a variety of basic helix-loop-helix (bHLH) genes responsible for the positive and negative regulation of neuronal differentiation of undifferentiated neural stem cells [43]. These included activator (Mash1, Math3 and NeuroD1) and repressor (Hes1 and Hes5) types of bHLH factors. In undifferentiated stable EV transfectants cultured for 4 days, marked mRNA expression was seen for Mash1, NeuroD1 and Hes1, but not for Math3 or Hes5 (Fig. 4A). In undifferentiated two clones of stable GlnT transfectants, drastic upregulation was invariably found in mRNA expression of Math3 and Hes5, in addition to Mash1 and NeuroD1 (Fig. 4B). However, *Hes1* mRNA was similarly expressed in undifferentiated P19 cells irrespective of stable overexpression of GlnT, in contrast to other bHLH proneural genes examined.

### NeuroD1 Promoter Activity

To confirm the upregulation of the activator type NeuroD1 mRNA expression, cells were transfected with a luciferase reporter plasmid linked to the NeuroD1 promoter from –1000 bp to +11 bp upstream, followed by culture for 4 days under floating conditions. A drastic increase was similarly induced in luciferase activity in two different clones of stable GlnT transfectants compared to stable EV transfectants (Fig. 5A). However, no significant change was found in luciferase activity with deletion promoter constructs of either −366/+11 bp or −112/+11 bp upstream in stable GlnT transfectants.

Since the green tea ingredient theanine is a structural analog of Gln (Fig. 5B) with a property to strongly inhibit [^3^H]Gln incorporation mediated by GlnT in cultured rat neurons and astroglia [22], pharmacological profiles of this inhibitor were next investigated with the NeuroD1 promoter activity in stable GlnT transfectants *in vitro*. In control stable EV transfectants, indeed, sustained exposure to 100 µM theanine for 4 days more than doubled luciferase activity with the full length promoter plasmid of –1000/+11 bp upstream (Fig. 5C). In two different clones of stable GlnT transfectants, by contrast, no significant further promotion was found in the drastically-elevated luciferase activity even after sustained exposure to 100 µM theanine under similar experimental conditions.

### Pharmacological Profiles of Theanine in Stable GlnT Transfectants

Sustained exposure to 100 µM theanine for 4 days markedly increased the size of aggregates composed of clustered cells in control stable EV transfectants cultured under floating conditions in line with the increased NeuroD1 promoter activity, while theanine failed to further enlarge the increased size of aggregates in both G13 and G17 clones of stable GlnT transfectants (Fig. 6A). Repetition and quantification of these experiments clearly confirmed the failure of theanine to additionally increase the size of aggregates composed of clustered cells in both G13 and G17 clones of stable GlnT transfectants, along with a significant increase by theanine in the aggregates size in control stable EV transfectants (Fig. 6B). Similarly, a significant increase was seen in MTT reduction in control stable EV transfectants exposed to theanine for 4 days, but not in the two clones of stable GlnT transfectants cultured with 100 µM theanine for 4 consecutive days (Fig. 6C). Prior exposure to theanine for 4 days significantly increased the number of cells immunoreactive for MAP2 in stable EV transfectants when determined 2 days after dispersion, while no significant further increase was seen in the number of MAP2-positive cells in the G13 clone of stable GlnT transfectants (Fig. 6D).

In order to evaluate the possible similarity between GlnT overexpression and theanine exposure, undifferentiated P19 cells were cultured with ATRA for 3 to 4 days in the presence of theanine at different concentrations up to 100 µM under floating conditions. Irrespective of the culture period, indeed, sustained exposure to theanine at 100 µM significantly increased the aggregate size (Fig. 7A) and MTT reduction (Fig. 7B) determined as indices of cellular proliferation activity. Theanine at 100 µM was also effective in significantly increasing mRNA expression of NeuroD1 in stable EV transfectants cultured for 4 days (Fig. 7C).

## Discussion

The essential importance of the present findings is that stable overexpression of GlnT ( = slc38a1) led to marked increases in both aggregates formation and subsequent differentiation into MAP2-positive cells in undifferentiated pluripotent P19 cells. The present drastic upregulation of both activator and repressor types of bHLH proneural genes gives rise to an idea that particular bHLH factors would be responsible for the promotion of both proliferation for self-replication and neuronal differentiation in stable GlnT transfectants. For example, the activator type bHLH factor NeuroD1 is shown to be essential for the survival and neuronal differentiation of neural stem cells during adult neurogenesis [44], [45]. Microarray gene analysis identifies NeuroD1 as a gene highly correlated with the terminal differentiation into neurons during postnatal and adult neurogenesis [46]. *In vivo* overexpression of NeuroD1 leads to rapid appearance of cells with morphological and molecular features of matured neurons, while knockdown by shRNA of NeuroD1 results in marked inhibition of terminal neuronal differentiation [46].

In Mash1-null mice, moreover, a severe loss is particularly seen for neuronal precursors, together with disappearance of the Notch signaling target Hes5, in the ventral telencephalon enriched of Mash1 during neurogenesis [47]. Notch signaling is shown to mediate a cell-cell interaction for the maintenance of dividing cells and subsequent generation of different progeny cell lineages in a manner dependent on the expression of the repressor type bHLH genes Hes1 and Hes5, which antagonize the activator type neuronal bHLH genes such as Mash1 [48]. However, both Hes1 and Hes5 are essential Notch effectors in negatively regulating mammalian neuronal differentiation [49]. Taken together, the present drastic upregulation of both activator and repressor types of bHLH proneural genes would be at least in part involved in molecular mechanisms underlying the promotion of both proliferation and neuronal differentiation in pluripotent P19 cells with stable overexpression of GlnT. To our knowledge, this is the first direct demonstration of promotion of both proliferation and neuronal differentiation through a mechanism relevant to upregulation of activator and repressor types of bHLH proneural genes in undifferentiated pluripotent P19 cells with stable overexpression of GlnT.

Nevertheless, the exact signaling mechanism from stable overexpression of GlnT to upregulation of bHLH proneural genes is not clarified so far. One possible but hitherto unproven speculation is that the intracellular Gln level would be at least in part responsible for the promotion of transactivation of particular target genes including bHLH factors in the nucleus. For example, Gln is shown to inhibit the activation by arginine and leucine of the mammalian target of rapamycin (mTOR) signaling in cultured rat intestinal epithelial cells [50]. In cultured Jarkat cells, mTOR activation occurs only in the presence of an extracellular amino acid complement with Gln as an obligatory component [51]. Increased intracellular Gln levels lead to promotion of the influx of extracellular essential amino acids, such as leucine, in exchange for the efflux of intracellular Gln, which consequently results in activation of the mTOR signaling pathway required for protein translation, cell growth and deteriorated autophagy toward the facilitation of cell growth and proliferation [52]. Moreover, mTOR signals are shown to regulate the neural progenitor status through a mechanism associated with upregulation of Hes5 and Pax6 expression in pluripotent P19 cells cultured with ATRA [53]. These previous findings all give rise to an idea that stable overexpression of GlnT would lead to activation of the mTOR signaling pathway responsible for subsequent promotion of gene transactivation of particular activator and repressor bHLH factors in neural progenitors. The exact mechanism as well as the physiological significance, however, remains to be elucidated in future studies.

One of the interesting findings obtained in this study is that the Gln analog theanine ( = γ-glutamylethylamide) similarly promoted both proliferation and neuronal differentiation in P19 cells with stable EV transfection without further stimulating both activities in stable GlnT transfectants. Theanine is shown to protect hippocampal CA1 pyramidal neurons from delayed neuronal cell death in gerbils with bilateral forebrain global ischemia [25], while theanine is a poor inhibitor of ligand binding to three different ionotropic receptor subtypes of the excitatory amino acid neurotransmitter glutamate in rat cortical synaptic membranes [26]. A structural evaluation clearly reveals a higher similarity of theanine to Gln rather than glutamate with regard to the amide moiety, suggesting that theanine could elicit its neuroprotective action through a mechanism relevant to Gln rather than glutamate in the brain. Theanine is metabolized by phosphate-independent glutaminase responsible for the degradation of Gln in rat kidney, but not by phosphate-dependent glutaminase enriched in the brain [54]. In our previous study [22], indeed, theanine strongly inhibits [^3^H]Gln incorporation sensitive to MeAIB without affecting [^3^H]glutamate incorporation in rat brain synaptosomal fractions. Moreover, [^3^H]theanine is incorporated into cultured rat brain neurons and astroglia in a Gln-sensitive manner [22]. These previous findings together with the present data give support to the proposal that theanine would promote both proliferation and neuronal differentiation through a mechanism common to stable GlnT overexpression in undifferentiated pluripotent P19 cells. From this point of view, it should be emphasized that stable overexpression of GlnT drastically facilitated the promoter activity of NeuroD1 endowed to activate neuronal differentiation with a concomitant failure of theanine to further stimulate the elevated promoter activity in undifferentiated pluripotent cells. By taking into consideration the relatively high effective concentration, however, it is unlikely that daily intake of green tea would positively regulate the proliferation for self-replication and the differentiation into neurons of neural stem cells locally expressed in particular brain regions in human beings *in vivo*. The possibility that theanine would be a future target molecule for the discovery and development of drugs useful for the prophylaxis and/or treatment of different neurodegenerative and neuropsychiatric diseases, however, is not ruled out so far.

### Conclusion

It thus appears that GlnT plays a role as a modulator of both proliferation and neuronal differentiation in neural progenitor cells before commitment during neurogenesis, in addition to a role as a supplier of the neurotransmitters GABA and glutamate in neurons, in the brain. Modulation of the functionality of GlnT would be thus of a great benefit for the regeneration and supplementation without surgical implantations of progenitor cells in patients with a variety of brain diseases relevant to neuronal and/or astroglial dysfunctions in a particular situation.

## References

[1] Schousboe A, Hertz L, Svenneby G, Kvamme E (1979) Phosphate activated glutaminase activity and glutamine uptake in primary cultures of astrocytes. J Neurochem 32: 943–950. 430071 10.1111/j.1471-4159.1979.tb04579.x

[2] Broer S (2002) Adaptation of plasma membrane amino acid transport mechanisms to physiological demands. Pflugers Arch 444: 457–466. 12136264 10.1007/s00424-002-0840-y

[3] Dolinska M, Zablocka B, Sonnewald U, Albrecht J (2004) Glutamine uptake and expression of mRNA’s of glutamine transporting proteins in mouse cerebellar and cerebral cortical astrocytes and neurons. Neurochem Int 44: 75–81. 12971909 10.1016/s0197-0186(03)00123-2

[4] Balcar VJ, Johnston GAR (1975) High affinity uptake of L-glutamine in rat brain slices. J Neurochem 24: 875–879. 167126 10.1111/j.1471-4159.1975.tb03650.x

[5] Reimer RJ, Chaudhry FA, Gray AT, Edwards RH (2000) Amino acid transport system A resembles system N in sequence but differs in mechanism. Proc Natl Acad Sci USA 97: 7715–7720. 10859363 10.1073/pnas.140152797 PMC16610

[6] Sugawara M, Nakanishi T, Fei YJ, Huang W, Ganapathy ME, et al (2000) Cloning of an amino acid transporter with functional characteristics and tissue expression pattern identical to that of system A. J Biol Chem. 275: 16473–16477. 10.1074/jbc.C000205200 10747860

[7] Varoqui H, Zhu H, Yao D, Ming H, Erickson JD (2000) Cloning and functional identification of a neuronal glutamine transporter. J Biol Chem 275: 4049–4054. 10660562 10.1074/jbc.275.6.4049

[8] Broer A, Brookes N, Ganapathy V, Dimmer KS, Wagner CA, et al (1999) The astroglial ASCT2 amino acid transporter as a mediator of glutamine efflux. J Neurochem 73: 2184–2194. 10537079

[9] Utsunomiya-Tate N, Endou H, Kanai Y (1996) Cloning and functional characterization of a system ASC-like Na^+^-dependent neutral amino acid transporter. J Biol Chem 271: 14883–14890. 8662767 10.1074/jbc.271.25.14883

[10] Chaudhry FA, Reimer RJ, Krizaj D, Barber D, Storm-Mathisen J, et al (1999) Molecular analysis of system N suggests novel physiological roles in nitrogen metabolism and synaptic transmission. Cell 99: 769–780. 10619430 10.1016/s0092-8674(00)81674-8

[11] Chillaron J, Roca R, Valencia A, Zorzano A, Palacn M (2001) Heteromeric amino acid transporters: biochemistry, genetics, and physiology. Am J Physiol Renal Physiol 281: F995–F1018. 11704550 10.1152/ajprenal.2001.281.6.F995

[12] Wagner CA, Lang F, Broer S (2001) Function and structure of heteromeric amino acid transporters. Am J Physiol Cell Physiol 281: C1077–C1093. 11546643 10.1152/ajpcell.2001.281.4.C1077

[13] Nagaraja TN, Brookes N. 1996. Glutamine transport in mouse cerebral astrocytes. J Neurochem 66: 1665–1674. 8627324 10.1046/j.1471-4159.1996.66041665.x

[14] Albers A, Broer A, Wagner CA, Setiawan I, Lang PA, et al (2001) Na^+^ transport by the neural glutamine transporter ATA1. Pflugers Arch 443: 92–101. 11692272 10.1007/s004240100663

[15] Chaudhry FA, Schmitz D, Reimer RJ, Larsson P, Gray AT, et al (2002) Glutamine uptake by neurons: interaction of protons with system a transporters. J Neurosci 22: 62–72. 11756489 10.1523/JNEUROSCI.22-01-00062.2002 PMC6757603

[16] Heckel T, Broer A, Wiesinger H, Lang F, Broer S (2003) Asymmetry of glutamine transporters in cultured neural cells. Neurochem Int 43: 289–298. 12742071 10.1016/s0197-0186(03)00014-7

[17] Deitmer JW, Broer A, Broer S (2003) Glutamine efflux from astrocytes is mediated by multiple pathways. J Neurochem 87: 127–135. 12969260 10.1046/j.1471-4159.2003.01981.x

[18] Melone M, Quagliano F, Barbaresi P, Varoqui H, Erickson JD, et al (2004) Localization of the glutamine transporter SNAT1 in rat cerebral cortex and neighboring structures, with a note on its localization in human cortex. Cereb Cortex 14: 562–574. 15054072 10.1093/cercor/bhh018

[19] Ogura M, Nakamichi N, Takano K, Oikawa H, Kambe Y, et al (2006) Functional expression of a glutamine transporter responsive to down-regulation by lipopolysaccharide through reduced promoter activity in cultured rat neocortical astrocytes. J Neurosci Res 83: 1447–1460. 16583402 10.1002/jnr.20855

[20] Ogura M, Taniura H, Nakamichi N, Yoneda Y (2007) Upregulation of the glutamine transporter through transactivation mediated by cAMP/protein kinase A signals toward exacerbation of vulnerability to oxidative stress in rat neocortical astrocytes. J Cell Physiol 212: 375–385. 17323379 10.1002/jcp.21031

[21] Hatanaka T, Huang W, Martindale RG, Ganapathy V (2001) Differential influence of cAMP on the expression of the three subtypes (ATA1, ATA2, and ATA3) of the amino acid transport system A. FEBS Lett. 505: 317–320. 10.1016/s0014-5793(01)02848-4 11566196

[22] Kakuda T, Hinoi E, Abe A, Nozawa A, Ogura M, et al (2008) Theanine, an ingredient of green tea, inhibits [^3^H]glutamine transport in neurons and astroglia in rat brain. J Neurosci Res 86: 1846–1856. 18293419 10.1002/jnr.21637

[23] Kakuda T, Nozawa A, Unno T, Okamura N, Okai O (2000) Inhibiting effects of theanine on caffeine stimulation evaluated by EEG in the rat. Biosci Biotechnol Biochem 64: 287–293. 10737183 10.1271/bbb.64.287

[24] Yokogoshi H, Kato Y, Sagesaka YM, Takihara-Matsuura T, Kakuda T, Takeuchi N (1995) Reduction effect of theanine on blood pressure and brain 5-hydroxyindoles in spontaneously hypertensive rats. Biosci Biotechnol Biochem 59: 615–618. 7539642 10.1271/bbb.59.615

[25] Kakuda T, Yanase H, Utsunomiya K, Nozawa A, Unno T, et al (2000) Protective effect of γ-glutamylethylamide (theanine) on ischemic delayed neuronal death in gerbils. Neurosci Lett 289: 189–192. 10961661 10.1016/s0304-3940(00)01286-6

[26] Kakuda T, Nozawa A, Sugimoto A, Niino H (2002) Inhibition by theanine of binding of [^3^H]AMPA, [^3^H]kainate, and [^3^H]MDL 105,519 to glutamate receptors. Biosci Biotechnol Biochem 66: 2683–2686. 12596867 10.1271/bbb.66.2683

[27] Gage FH, Coates PW, Palmer TD, Kuhn HG, Fisher LJ, et al (1995) Survival and differentiation of adult neuronal progenitor cells transplanted to the adult brain. Proc Natl Acad Sci USA 92: 11879–11883. 8524867 10.1073/pnas.92.25.11879 PMC40506

[28] Suhonen JO, Peterson DA, Ray J, Gage FH (1996) Differentiation of adult hippocampus-derived progenitors into olfactory neurons *in vivo* . Nature 383: 624–627. 8857538 10.1038/383624a0

[29] Doetsch F, Caille I, Lim DA, Garcia-Verdugo JM, Alvarez-Buylla A (1999) Subventricular zone astrocytes are neural stem cell in the adult mammalian brain. Cell 97: 703–716. 10380923 10.1016/s0092-8674(00)80783-7

[30] Johansson CB, Momma S, Clarke DL, Risling M, Lendahl U, et al (1999) Identification of a neural stem cell in the adult mammalian central nervous system. Cell 96: 25–34. 9989494 10.1016/s0092-8674(00)80956-3

[31] Temple S, Alvarez-Buylla A (1999) Stem cells in the adult mammalian central nervous system. Curr Opin Neurobiol 9: 135–141. 10072370 10.1016/s0959-4388(99)80017-8

[32] Ogura M, Takarada T, Nakamichi N, Kawagoe H, Sako A, et al (2011) Exacerbated vulnerability to oxidative stress in astrocytic C6 glioma cells with stable overexpression of the glutamine transporter slc38a1. Neurochem Int 58: 504–511. 21219957 10.1016/j.neuint.2011.01.007

[33] Hinoi E, Fujimori S, Wang L, Hojo H, Uno K, et al (2006) Nrf2 negatively regulates osteoblast differentiation via interfering with Runx2 dependent transcriptional activation. J Biol Chem 281: 18015–18024. 16613847 10.1074/jbc.M600603200

[34] Nakamura Y, Takarada T, Kodama A, Hinoi E, Yoneda Y (2009) Predominant promotion by tacrolimus of chondrogenic differentiation to proliferating chondrocytes. J Pharmacol Sci 109: 413–423. 19270431 10.1254/jphs.08315fp

[35] Fukumori R, Nakamichi N, Takarada T, Kambe Y, Matsushima N, et al (2010) Inhibition by 2-methoxy-4-ethylphenol of Ca^2+^ influx through acquired and native N-methyl-D-aspartate receptor channels. J Pharmacol Sci 112: 273–281. 20168047 10.1254/jphs.09294fp

[36] Jones-Villeneuve EM, Rudnicki MA, Harris JF, McBurney MW (1983) Retinoic acid-induced neural differentiation of embryonic carcinoma cells. Mol Cell Biol 3: 2271–2279. 6656766 10.1128/mcb.3.12.2271-2279.1983 PMC370098

[37] Mummery CL, Feijen A, van der Saag PT, van den Brink CE, de Laat SW (1985) Clonal variants of differentiated P19 embryonal carcinoma cells exhibit epidermal growth factor receptor kinase activity. Dev Biol 109: 402–410. 2987069 10.1016/0012-1606(85)90466-x

[38] Uno K, Takarada T, Nakamura Y, Fujita H, Hinoi E, et al (2011) A negative correlation between expression profiles of runt-related transcription factor-2 and cystine/glutamate antiporter xCT subunit in ovariectomized mouse bone. J Pharmacol Sci 115: 309–319. 21325781 10.1254/jphs.10310fp

[39] Yoneyama M, Fukui M, Nakamichi N, Kitayama T, Taniura H, et al (2007) Activation of GABA_A_ receptors facilitates astroglial differentiation induced by ciliary neurotrophic factor in neural progenitors isolated from fetal rat brain. J Neurochem 100: 1667–1679. 17212695 10.1111/j.1471-4159.2006.04322.x

[40] Fukui M, Ozawa S, Nakamichi N, Nakazato R, Takarada T, et al (2011) Gradual downregulation of protein expression of the partner GABA_B_R2 subunit during postnatal brain development in mice defective of GABA_B_R1 subunit. J Pharmacol Sci 115: 45–55. 21160134 10.1254/jphs.10254fp

[41] Takarada T, Yoneda Y. 2009. Transactivation by runt related factor-2 of matrix metalloproteinase-13 in astrocytes. Neurosci Lett 451: 99–104. 19121369 10.1016/j.neulet.2008.12.037

[42] Takarada T, Tamaki K, Takumi T, Ogura M, Ito Y, et al (2009) A protein-protein interaction of stress-responsive myosin VI endowed to inhibit neural progenitor self-replication with RNA binding protein, TLS, in murine hippocampus. J Neurochem 110: 1457–1468. 19558455 10.1111/j.1471-4159.2009.06225.x

[43] Bertrand N, Castro DS, Guillemot F (2002) Proneural genes and the specification of neural cell types. Nat Rev Neurosci 3: 517–530. 12094208 10.1038/nrn874

[44] Kuwabara T, Hsien J, Muotri A, Yeo G, Warashina M, et al (2009) Wnt-mediated activation of NeuroD1 and retro-elements during adult neurogenesis. Nature Neurosci 12: 1097–1105. 19701198 10.1038/nn.2360 PMC2764260

[45] Gao Z, Ure K, Ables JL, Lagace DC, Nave KA, et al (2009) Neurod1 is essential for the survival and maturation of adult-born neurons. Nature Neurosci 12: 1090–1092. 19701197 10.1038/nn.2385 PMC3365543

[46] Boutin C, Hardt O, de Chevigny A, Core N, Goebbels S, et al (2010) NeuroD1 induces terminal neuronal differentiation in olfactory neurogenesis. Proc Natl Acad Sci USA 107: 1201–1206. 20080708 10.1073/pnas.0909015107 PMC2824315

[47] Casarosa S, Fode C, Guillemot F (1999) Mash1 regulates neurogenesis in the ventral telencephalon. Development 126: 525–534. 9876181 10.1242/dev.126.3.525

[48] Kageyama R, Ohtsuka T (1999) The Notch-Hes pathway in mammalian neural development. Cell Res 9: 179–188. 10520600 10.1038/sj.cr.7290016

[49] Ohtsuka T, Ishibashi M, Gradwohl G, Nakanishi S, Guillemot F, et al (1999) Hes1 and Hes5 as notch effectors in mammalian neuronal differentiation. EMBO J 18: 2196–2207. 10205173 10.1093/emboj/18.8.2196 PMC1171303

[50] Nakajo T, Yamatsuji T, Ban H, Shigemitsu K, Haisa M, et al (2005) Glutamine is a key regulator for amino acid-controlled cell growth through the mTOR signaling pathway in rat intestinal epithelial cells. Biochem Biophys Res Commun 326: 174–180. 15567168 10.1016/j.bbrc.2004.11.015

[51] Fumarola C, La Monica S, Guidotti GG (2005) Amino acid signaling through the mammalian target of rapamycin (mTOR) pathway: Role of glutamine and of cell shrinkage. J Cell Physiol 204: 155–165. 15605414 10.1002/jcp.20272

[52] Nicklin P, Bergman P, Zhang B, Triantafellow E, Wang H, et al (2009) Bidirectional transport of amino acids regulates mTOR and autophagy. Cell 136: 521–534. 19203585 10.1016/j.cell.2008.11.044 PMC3733119

[53] Endo M, Antonyak MA, Cerione RA (2009) Cdc42-mTOR signaling pathway controls Nes5 and Pax6 expression in retinoic acid-dependent neural differentiation. J Biol Chem 284: 5107–5118. 19097998 10.1074/jbc.M807745200 PMC3837441

[54] Tsuge H, Sano S, Hayakawa T, Kakuda T, Unno T (2003) Theanine, gamma-glutamylethylamide, is metabolized by renal phosphate-independent glutaminase. Biochim Biophys Acta 1620: 47–53. 12595072 10.1016/s0304-4165(02)00504-4

